# The Effectiveness of Antiviral Treatments for Patients with HBeAg-Positive Chronic Hepatitis B: A Bayesian Network Analysis

**DOI:** 10.1155/2018/3576265

**Published:** 2018-09-12

**Authors:** Zhang Hao, Zhu Biqing, Yang Ling, Zeng Wenting

**Affiliations:** ^1^Department of Infectious Diseases, The First Affiliated Hospital of Guangzhou Medical University, Guangzhou Medical University, Yanjiang Road 151, Guangzhou 510120, China; ^2^Guangzhou Kingmed Center for Clinical Laboratory Co., Ltd., Guangzhou International Biological Island, Spiral Three Road 10, Guangzhou 510330, China

## Abstract

This network analysis is to determine the most effective treatment in HBeAg-positive patients. PubMed databases were searched for randomized controlled trials. Bayesian network meta-analysis was used to calculate the pairwise hazard ratios, 95% credible intervals, and ranking of surrogate outcomes. 9 studies were identified. The results show that NA add-on PEG IFN might be a better antiviral approach for HBeAg-positive patients in end point of treatment, with a comparable results of nucleoside/nucleotide analogs (NA), PEG IFN, PEG IFN add-on NA, PEG IFN combined NA, and PEG IFN combined placebo in alanine aminotransferase (ALT) normalization and HBV DNA undetectable. Cumulative probabilities of being the most efficacious treatment were NA add-on PEG IFN (30%) for HBeAg loss. The second efficacious (23%) is HBeAg seroconversion. This network analysis shows that NA add-on PEG IFN might be a better antiviral approach for HBeAg-positive patients in end point of treatment. But the long-term efficiency should be further determined.

## 1. Introduction

Hepatitis B virus (HBV) infection is one of the most common persistent viral infections in human beings. Over 240 million people worldwide are estimated to currently be chronically infected with HBV [1]. Chronic infection of HBV leads to serious medical complications, such as cirrhosis, hepatocellular carcinoma, and liver failure. The number of deaths from liver cirrhosis, liver cancer, and acute hepatitis due to HBV infection has greatly increased [2]. This highlights the need for effective treatments for chronic hepatitis B (CHB).

Currently, two sets of treatments are available for the treatment of HBV infection, which are the nucleoside/nucleotide analogs (NA) or PEGylated interferon (PEG IFN)[3]. With currently available treatment, HBsAg loss is uncommon; approximately 3–5% of the patients treated with PEG IFN, and 0 to 3% of patients treated with NA, lose HBsAg [4]. In order to improve response to antiviral treatment, different studies have used add-on therapy in various combinations. These include the simultaneous administration of the two drugs in naïve patients, “add-on” or “switch to” strategies in patients already on therapy [5–7]. However, the results are inconsistent. Therefore, we are performed the evidence base by conducting a network analyses of all published trials of different antiviral treatments (monotherapy, combination, adding on, or switching). Our aim is to determine which treatment is the most effective in treating CHB patients by analyzing surrogate outcomes in CHB.

## 2. Methods

### 2.1. Eligibility Criteria

The eligibility criteria for inclusion into this network analyses are studies involving adults with HBeAg-positive CHB in randomized, controlled trials (RCTs) that investigated a combination of the following therapies (such as monotherapy, combination, add-on, or switching): placebo (PLA), lamivudine (LAM), adefovir (ADV), entecavir (ETV), lamivudine (LdT), tenofovir (TDF), and PEG IFN. The following exclusion criteria were excluded in this study: (1) non-RCTs; (2) coinfection with hepatitis A, C, D, or E, cytomegalovirus, or HIV; (3) patients who were children; (4) patients who were not with HBeAg-positive; (5) patients who had liver failure, HCC, or other liver related complications caused by autoimmune diseases, drugs, or alcoholism.

### 2.2. Literature Search

PubMed were searched for potential references along with citation searching of relevant articles. The search was limited to English language publications. The original review conducted up to 15th January 2018. The search was conducted using the key words ‘HBV or hepatitis B or CHB' and ‘IFN or interferon' and ‘random*∗*'. Potentially relevant papers were reviewed by two authors (Zhang H and Zhu BQ) and a third author (Yang L) addressed disagreements. Papers from the original review were also retrieved and reviewed. Meeting abstracts and unpublished data were not included.

### 2.3. Efficacy Measures

Efficacy was evaluated based on the following criteria: alanine aminotransferase (ALT) normalization: ALT levels < 40 IU/ml; undetectable HBV DNA: HBV DNA levels < 1,000 copies/ml or less; HBeAg loss; HBeAg seroconversion: HBeAg loss and occurrence of HBeAb at the end of treatment (EOT).

### 2.4. Data Extraction

Data extraction was carried out by two independent reviewers (Zhang H and Zhu BQ). We recorded the following for each study: (1) trial characteristics (the first author's name, published year, country of study, sum of each group, and quality of RCT); (2) patient characteristics (mean age, ethnicity of patients); (3) the details of each regimen (i.e., the antiviral drug used and treatment duration); and (4) observation time and outcomes. We contacted the authors of the eligible publications that had inadequate information; if effective data were still not obtained, those papers were excluded. All the data were reviewed to eliminate duplicate reports of the same trial.

### 2.5. Assessment for Risk of Bias

We used the Jadad scale to evaluate the quality of the RCTs [15]. The quality of each trial was assessed independently by two study investigators (Zhang H and Zhu BQ). Discrepancies were resolved by discussion with a third person (Zeng WT).The Jadad scale was used to score the methodological quality of RCTs based on the following items: randomization (0−1 points), blinding (0−1 points), and dropouts and withdrawals (0−1 point).

### 2.6. Statistical Analysis

First, we conducted pairwise meta-analyses to synthesize studies comparing the same pair of treatments with STATA 11.0 software. The results were reported as pooled hazard ratios (HRs) with the corresponding 95% confidence interval (CI). Regression analyses were performed to estimate funnel plot asymmetry. Heterogeneity was explored by the chi-squared test and I^2^ test with significance limit set at a P value of 0.10.

Second, we built a fixed-effects network within a Bayesian framework, which were burned-in for 5000 Markov Chain Monte Carlo iterations and convergence was based on the Gelman-Rubin-Brooke statistic. A further 25 000 iterations were run and the sampled values were used to estimate response probabilities and HRs. The analysis was performed using in Gemtc software. We networked the translated binary outcomes within studies and specified the relations among the HRs across studies making different comparisons. This method combined direct and indirect evidence for any given pair of treatments. We used P < 0.05 and 95% CIs to assess significance.

## 3. Results

### 3.1. Study Characteristics

**Figure 1:**
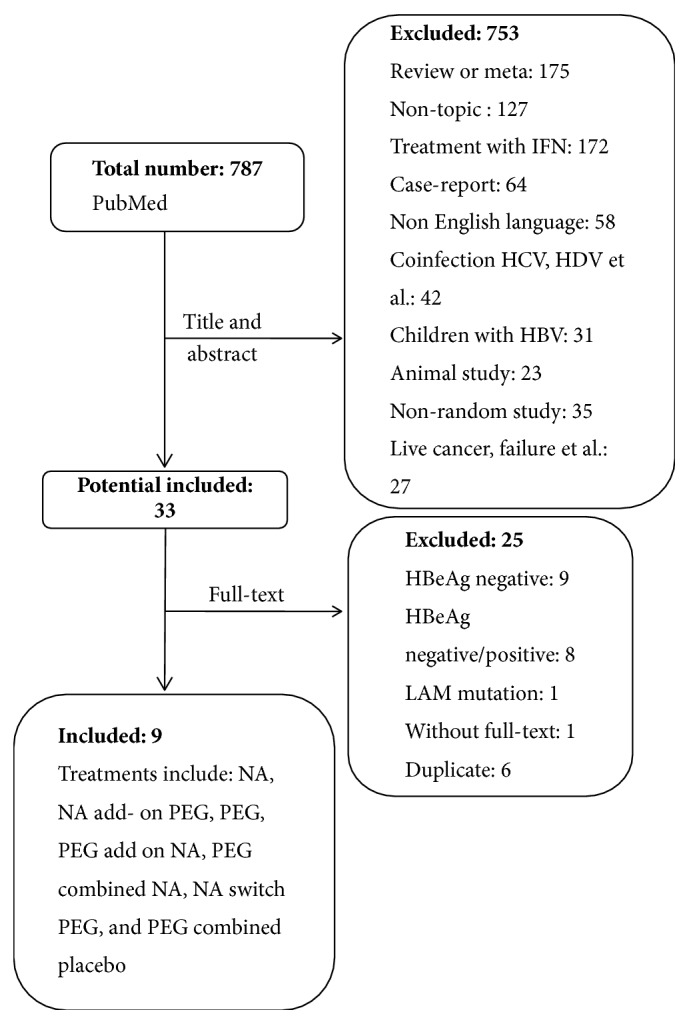
Flow chart of study selection and exclusion.

Figure 1 describes the literature search and exclusion of studies. In total, 9 studies were identified (2 023 patients). We identified 7 trials were designed as two-arm trials analyzing [6–9, 11, 13, 14], whereas the other 2 were three-arm trials [10, 12] (Table 1). Of these, 590 received NA (29.2%), 129 received NA add-on PEG IFN (6.4%), 237 received PEG IFN (11.7%), 137 received PEG IFN add-on NA (6.8%), 431 received PEG IFN combined NA (21.3%), 92 received NA switch PEG IFN (4.5%), and 407 received PEG IFN combined Placebo (20.1%).

### 3.2. Direct Meta-Analyses

*ALT Normalization.* All the included trials reported the rate of ALT normalization. The significant difference was found in the treatment of NA versus NA switch PEG IFN (Pooled HR 1.392, p=0.019) and NA versus PEG IFN combined PLA (Pooled HR 1.367, p=0.002). That means patients with NA treatment could achieve high rate of ALT normalization compared with the above two treatments. However, no significant differences were found among the treatments: NA versus NA add-on PEG IFN, PEG IFN versus PEG IFN add-on NA, PEG IFN versus NA, PEG IFN add-on NA versus NA, PEG IFN versus PEG IFN combined NA, NA vs. PEG IFN combined NA, and PEG IFN combined versus PEG IFN combined PLA (Table 2). As the combined analyses were no more than three studies, the heterogeneity cannot be performed.

*HBV Undetectable.* All the included trials also reported the rate of HBV undetectable. The significant difference was found in the treatment of PEG IFN versus PEG IFN combined NA (Pooled HR 0.518, p=0.048), NA versus PEG IFN combined NA (Pooled HR 0.698, p<0.001), NA versus PEG IFN combined PLA (Pooled HR 1.417, p=0.01), and PEG IFN combined NA versus PEG IFN combined PLA (Pooled HR 2.153, p<0.001). No significant differences were found among the treatments: NA versus NA add-on PEG IFN, PEG IFN versus PEG IFN add-on NA, PEG IFN versus NA, PEG IFN add-on NA versus NA, and NA versus NA switch PEG IFN (Table 2). As the combined analyses were no more than three studies, the heterogeneity cannot be performed.

*HBeAg Loss and Seroconversion*. All the included trials also reported the rate of HBeAg seroconversion. Seven of them reported the rate of HBeAg loss. No significant differences were in all the treatments under the direct analyses (Table 2).

### 3.3. Network Meta-Analyses

**Figure 2:**
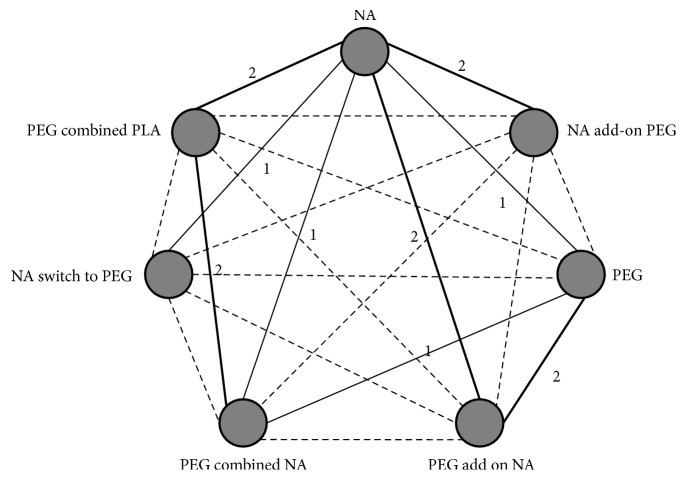
Network of trial comparisons for NA, NA add-on PEG IFN, PEG IFN, PEG IFN add-on NA, PEG IFN combined NA, NA switch to PEG IFN, PEG IFN combined PLA. NA, nucleoside/nucleotide analogs; PEG IFN, pegylated interferon; PLA, placebo. Numbers represent that number of direct comparisons available. Dashed lines indicate indirect treatment comparisons.

The structure of the network analysis is reported in Figure 2.  Table 3 summarizes the results of the network meta-analysis for ALT normalization, HBV undetectable, HBeAg loss, and seroconversion. The significant differences of ALT normalization were found in the treatment of NA (HR 9.68, 95% CI 2.59-41.22), PEG IFN (HR 9.00, 95% CI 1.83-51.79), PEG IFN add-on NA (HR 18.05, 95% CI 3.64-109.82), and PEG IFN combined NA (HR 6.76, 95% CI 1.49-43.48) versus NA switch PEG IFN, respectively. As expected, a significant low rate of HBV undetectable was found in the treatment of NA (HR 21.11, 95% CI 2.90-262.11), NA add-on PEG IFN (HR 36.56, 95% CI 3.27-567.15), PEG IFN (HR 17.12, 95% CI 1.47-248.25), PEG IFN add-on NA (HR 28.39, 95% CI 2.31-404.85), PEG IFN combined NA (HR 82.47, 95% CI 8.49-1406.85), and PEG IFN combined PLA (HR 13.49, 95% CI 1.21-236.27) versus NA switch PEG IFN, respectively.

**Figure 3:**
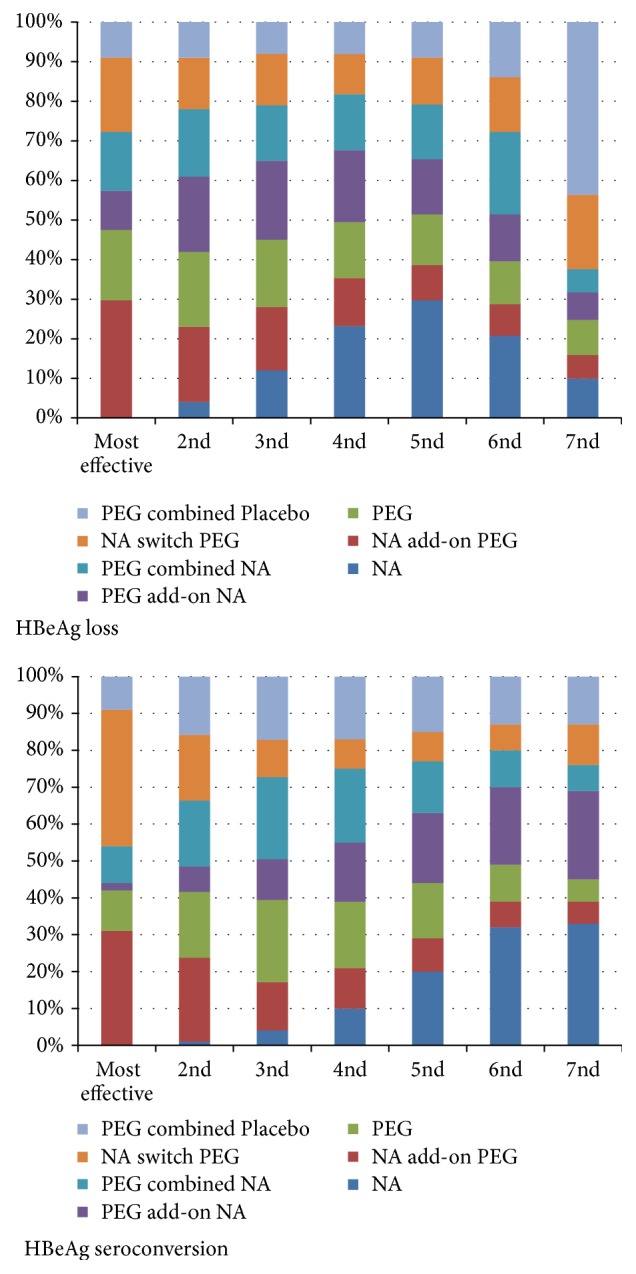
Rankogram reporting the probabilities of being the best treatment (reflective of the length in stacked bar for each drug in given column) in terms of HBeAg loss and HBeAg seroconversion.

As no significant differences of HBeAg loss and seroconversion existed in the treatments (Table 3), the rank probability to be the best treatment should be showed in Figure 3. Cumulative probabilities of being the most efficacious treatment were as follows: NA add-on PEG IFN (30%) for HBeAg loss and NA switch PEG IFN (37%) for HBeAg seroconversion. The followed approach is NA add-on PEG IFN for HBeAg seroconversion (23%). There was no significant inconsistency within the network meta-analysis.

## 4. Discussion

Conventional meta-analysis cannot compare the relative effect of one drug to another unless they were compared to each other in the same study. In network meta-analysis, multiple treatment comparisons for a specific disease, which were not compared to each other, can be made simultaneously through a common comparator treatment [16–19]. This network analysis of 9 clinical trials shows that NA add-on PEG IFN is more effective in HBeAg-positive patients based on the goal of loss of HBV DNA, loss of HBeAg, and development of anti-HBeAg antibodies. Also, to the best of our knowledge, this is the first study that provides both direct and indirect evidence in terms of comparative effectiveness of antiviral treatments (monotherapy, combination, adding on, or switching) by included RCTs studies.

Theoretically, a combined NA and PEG IFN approach may provide advantages by combining the potent antiviral effect of NA plus the immune modulation of IFN [5, 20]. However, the evidence of such a combined approach is lacking. In our network analysis, this combined approach was not better than other antiviral approaches in the ALT normalization, HBV undetectable, HBeAg loss, and HBeAg seroconversion, except in the treatment of NA switch PEG IFN. Also, this combined antiviral treatment costs more than other approaches.

Our network analysis showed that NA add-on PEG IFN might be a better antiviral approach for HBeAg-positive patients based on the HBV DNA undetectable, HBeAg loss, and HBeAg seroconversion. As we all know, All NAs are competitive inhibitors of the natural endogenous intracellular nucleotide, which means NAs are effective in suppressing HBV replication [21]. However, the challenge of antiviral therapy is to clear the HBV covalently closed circular DNA (cccDNA) pool. NA has been reported to reduce intrahepatic as well as serum cccDNA [22]. But it is unknown whether long-term NAs have a greater effect on HBV intrahepatic cccDNA decline. As reported, low quantitative hepatitis B surface antigen (qHBsAg) and HBV DNA were strong predictive stopping rule in HBV patients treated with PEG IFN [23]. Serum qHBsAg appears to be more strongly correlated with cccDNA levels in HBeAg-positive patients [24]. Thus, patients first with NA treatment achieved undetectable HBV DNA, followed by adding on PEG IFN which might get a better efficiency.

Our analyses have some strengths, including the use of an exhaustive search strategy, use of RCTs studies, and treatment comparisons by Bayesian networks. However, the results need to be interpreted with caution for the following reasons.

First, the initial treatment for patients with HBeAg-positive was different among the included studies. Table 1 has shown the results. Second, the criteria of included patients were different, and the heterogeneity cannot performed because no more studies were included. Third, most of the studies were performed in China. Forth, HBsAg loss and/or seroconversion which are the major end points of successful HBV therapy were not analyzed, because the data of HBsAg loss and/or seroconversion is limited in the included studies. Therefore, more clinical studies performed in different populations are necessary to access the generalizability of the results. Finally, the efficiency of the network study was based on the end point of treatments. The long-term efficiency should be further determined.

## 5. Conclusion

This network analysis shows that NA add-on PEG IFN might be a better antiviral approach for HBeAg-positive patients in end point of treatment. Studies of combination therapy with PEG IFN and NA are still ongoing in a large cohort of patients with a long-term follow-up, and it is possible that this add-on approach may be a future option that may be considered in individual patients, when more robust data will provide definitive evidence of efficacy and clinical benefits.

## Figures and Tables

**Table 1:** Main characteristics of studies included.

Study	Country	Year	Project	Initial Treatment	Include patients	End point	Randomized Treatment	Jada Scores
Chi H et al. [8]	Netherlands, China	2017	NCT01532843	≥12wk ETV or TDF	HBV DNA <2 000 IU/mL, ALT<5 times the upper limit of normal	ALT normalization, HBV DNA undectable, HBeAg loss and HBeAg seroconversion	NA, NA add-on PEG IFN	2

Sun J et al. [9]	China	2016	NCT01086085	24wk PEG IFN	Non early responders	ALT normalization, HBV DNA undectable, HBeAg loss and HBeAg seroconversion	PEG IFN, PEG IFN add- on NA	2

Brouwer WP et al. [6]	Europe, Asia	2015	NCT00877760	24wk ETV	ALT >1.3 times the upper limit of normal	ALT normalization, HBV DNA undectable, HBeAg loss and HBeAg seroconversion	NA, NA add-on PEG IFN	2

Xie Q et al. [10]	China	2014	NCT00614471	NA	HBV DNA ≥100 000 copies/mL, ALT >2 but ≤10 times the upper limit of normal	ALT normalization, HBV DNA undectable, HBeAg loss and HBeAg seroconversion	PEG IFN, NA and PEG IFN add-on NA	2

Liu YH et al. [11]	China	2014	-	NA	HBV DNA ≥100 000 copies/mL, ALT >2 but ≤10 times the upper limit of normal	ALT normalization, HBV DNA undectable, HBeAg loss and HBeAg seroconversion	PEG IFN, PEG IFN combined NA	1

Ning Q et al. [7]	China	2014	OSST	9-36 months ETV	HBV DNA <1000copies/mL, HBeAg<100PEIU/L	ALT normalization, HBV DNA undectable, HBeAg loss and HBeAg seroconversion	NA, NA switch PEG IFN	2

Lau GK et al. [12]	16 countries	2005	-	NA	HBsAg negative, HBV DNA ≥500 000 copies/mL, ALT >1 but ≤10 times the upper limit of normal	ALT normalization, HBV DNA undectable, HBeAg loss and HBeAg seroconversion	NA, PEG IFN combined PLA, and PEG IFN combined NA	3

Chan HL et al. [13]	Hong Kong	2005	-	-	HBV DNA ≥500 000 copies/mL, ALT >1.3 but ≤5 times the upper limit of normal	ALT normalization, HBV DNA undectable, and HBeAg seroconversion	PEG IFN add-on NA, NA	2

Janssen HL et al. [14]	15 countries	2005	-	-	ALT >2 times the upper limit of normal	ALT normalization, HBV DNA undectable, HBeAg loss and HBeAg seroconversion	PEG IFN combined NA, PEG IFN combined PLA	3

Note: NA, nucleoside/nucleotide analogs; PEG IFN, PEG IFNylated interferon; PLA, placebo; ETV, entecavir; TDF, tenofovir; wk, week

**Table 2:** The results of direct analysis estimates of efficacy (hazard ratio).

Treatment	Study	ALT normalization			HBV DNA undetectable			HBeAg loss			HBeAg seroconversion		
		HR	95% CI	p	HR	95% CI	p	HR	95% CI	p	HR	95% CI	p
NA vs. NA add-on PEG IFN	Chi H et al. [8] Brouwer WP et al. [6]	0.89	0.71-1.12	0.31	0.94	0.76-1.15	0.53	0.62	0.35-1.10	0.10	0.52	0.25-1.06	0.07
PEG IFN vs. PEG IFN add-on NA	Sun J et al. [9] Xie Q et al. [10]	0.83	0.64-1.07	0.16	0.82	0.61-1.11	0.21	0.76	0.54-1.09	0.13	0.83	0.55-1.25	0.37
PEG IFN vs. NA	Xie Q et al. [10]	1.03	0.67-1.59	0.88	1.07	0.73-1.55	0.74	0.78	0.48-1.29	0.34	0.96	0.49-1.86	0.89
PEG IFN add-on NA vs. NA	Xie Q et al. [10] Chan HL et al. [13]	1.14	0.89-1.47	0.30	0.74	0.53-1.03	0.08	1.21	0.85-1.73	0.29	1.31	0.86-1.99	0.21
PEG IFN vs. PEG IFN combined NA	Liu YH et al. [11]	0.89	0.48-1.64	0.70	**0.52**	**0.27-1.00**	**0.04**	NA	NA	NA	0.77	0.34-1.70	0.51
NA vs. NA switch PEG IFN	Ning Q et al. [7]	**1.39**	**106-1.84**	**0.02**	1.18	0.93-1.51	0.18	0.91	0.50-1.64	0.75	0.46	0.19-1.16	0.10
NA vs. PEG IFN combined NA	Lau GK et al. [12]	1.20	1.00-1.45	0.05	**0.70**	**0.58-0.85**	**<0.01**	0.84	0.62-1.14	0.27	0.88	0.64-1.22	0.45
NA vs. PEG IFN combined PLA	Lau GK et al. [12]	**1.37**	**1.12-1.67**	**<0.01**	**1.42**	**1.09-1.85**	**0.01**	1.34	0.95-1.90	0.09	0.80	0.58-1.10	0.17
PEG IFN combined NA vs. PEG IFN combined PLA	Lau GK et al. [12] Janssen H et al. [14]	1.20	1.00-1.43	0.05	**2.15**	**1.73-2.69**	**<0.01**	NA	NA	NA	1.12	0.72-1.75	0.82

Note: NA, nucleoside/nucleotide analogs; PEG IFN, PEG IFNylated interferon; PLA, placebo; HR, hazard ratio.

**Table 3:** League table presenting network meta-analysis estimates of efficacy (hazard ratio).

ALT normalization						
*NA*						
2.59 (0.70, 6.09)	*NA add-on PEG IFN *					
1.07 (0.41, 2.82)	0.42 (0.11, 2.32)	*PEG IFN *				
0.55 (0.22, 1.32)	0.21 (0.06, 1.07)	0.51 (0.21, 1.14)	*PEG IFN add-on NA *			
1.47 (0.46, 3.22)	0.56 (0.14, 2.52)	1.34 (0.39, 3.54)	2.64 (0.72, 7.73)	*PEG IFN combined NA*		
**9.68 (2.59, 41.22)**	3.80 (0.84, 27.37)	**9.00 (1.83, 51.79)**	**18.05 (3.64, 109.82)**	**6.76 (1.49, 43.48)**	*NA switch PEG IFN *	
2.49 (0.80, 6.49)	0.95 (0.26, 4.86)	2.32 (0.65, 7.42)	4.59 (1.26, 15.25)	1.71 (0.80, 4.32)	0.26 (0.04, 1.26)	*PEG IFN combined PLA*

HBV DNA undetectable						

*NA*						
0.59 (0.17, 2.27)	*NA add-on PEG IFN *					
1.25 (0.40, 5.05)	2.09 (0.38, 13.95)	*PEG IFN *				
0.74 (0.26, 3.21)	1.24 (0.25, 8.88)	0.59 (0.20, 2.04)	*PEG IFN add-on NA *			
0.26 (0.07, 0.91)	0.44 (0.07, 2.52)	0.21 (0.04, 0.74)	0.35 (0.06, 1.38)	*PEG IFN combined NA*		
**21.11 (2.90, 262.11)**	**36.56 (3.27, 567.15)**	**17.12 (1.47, 248.25)**	**28.69 (2.31, 404.85)**	**82.47 (8.49, 1406.85)**	*NA switch PEG IFN *	**13.49 (1.21, 236.27)**
1.64 (0.37, 6.51)	2.71 (0.36, 17.18)	1.30 (0.20, 5.62)	2.16 (0.29, 9.75)	6.23 (1.91, 19.45)		*PEG IFN + Placebo*

HBeAg loss						

*NA*						
0.52 (0.10, 2.73)	*NA add-on PEG IFN *					
0.63 (0.11, 3.29)	1.21 (0.11, 11.91)	*PEG IFN *				
0.68 (0.15, 2.75)	1.31 (0.14, 11.60)	1.09 (0.25, 4.64)	*PEG IFN add-on NA *			
0.75 (0.10, 5.48)	1.46 (0.11, 19.44)	1.19 (0.09, 17.27)	1.11 (0.10, 13.48)	*PEG IFN combined NA*		
0.81 (0.10, 6.95)	1.57 (0.11, 22.91)	1.31 (0.09, 21.36)	1.19 (0.10, 16.22)	1.08 (0.06, 21.17)	*NA switch PEG IFN *	
1.47 (0.08, 25.96)	2.83 (0.11, 76.01)	2.37 (0.08, 64.49)	2.15 (0.08, 52.38)	1.93 (0.26, 15.12)	1.80 (0.05, 62.85)	*PEG IFN combined PLA*

HBeAg seroconversion						

*NA*						
0.39 (0.08, 1.86)	*NA add-on PEG IFN *					
0.53 (0.13, 2.25)	1.34 (0.16, 12.44)	*PEG IFN *				
0.81 (0.21, 3.14)	2.09 (0.27, 16.73)	1.52 (0.39, 5.86)	*PEG IFN add-on NA *			
0.55 (0.12, 2.26)	1.40 (0.15, 12.65)	1.04 (0.20, 4.88)	0.69 (0.11, 3.79)	*PEG IFN combined NA*		
0.37 (0.04, 2.91)	0.94 (0.07, 13.35)	0.69 (0.05, 8.63)	0.45 (0.04, 5.23)	0.67 (0.05, 9.14)	*NA switch PEG IFN *	
0.62 (0.11, 3.00)	1.55 (0.16, 15.37)	1.17 (0.17, 7.39)	0.76 (0.10, 5.06)	1.12 (0.30, 4.32)	1.68 (0.10, 24.88)	*PEG IFN combined PLA*

Note: NA, nucleoside/nucleotide analogs; PEG IFN, PEG IFNylated interferon; PLA, placebo

## Data Availability

No data were used to support this study.

## References

[1] Ott J. J., Stevens G. A., Groeger J., Wiersma S. T. (2012). Global epidemiology of hepatitis B virus infection: new estimates of age-specific HBsAg seroprevalence and endemicity. *Vaccine*.

[2] Lozano R., Naghavi M., Foreman K. (2012). Global and regional mortality from 235 causes of death for 20 age groups in 1990 and 2010: a systematic analysis for the Global Burden of Disease Study 2010. *The Lancet*.

[3] Terrault N. A., Bzowej N. H., Chang K.-M., Hwang J. P., Jonas M. M., Murad M. H. (2016). AASLD guidelines for treatment of chronic hepatitis B. *Hepatology*.

[4] Liver EAFT (2012). EASL clinical practice guidelines: Management of chronic hepatitis B virus infection. *Journal of Hepatology*.

[5] Marcellin P., Ahn S. H., Ma X. (2016). Combination of tenofovir disoproxil fumarate and peginterferon *α*-2a increases loss of hepatitis B surface antigen in patients with chronic hepatitis B. *Gastroenterology*.

[6] Brouwer W. P., Xie Q., Sonneveld M. J. (2015). Adding pegylated interferon to entecavir for hepatitis B e antigen-positive chronic hepatitis B: a multicenter randomized trial (ARES study). *Hepatology*.

[7] Ning Q., Han M., Sun Y. (2014). Switching from entecavir to PegIFN alfa-2a in patients with HBeAg-positive chronic hepatitis B: a randomised open-label trial (OSST trial). *Journal of Hepatology*.

[8] Chi H., Hansen B. E., Guo S. (2017). Pegylated interferon Alfa-2b add-on treatment in hepatitis b virus envelope antigen-positive chronic hepatitis B patients treated with nucleos(t)ide analogue: A randomized, controlled trial (PEGON). *The Journal of Infectious Diseases*.

[9] Sun J., Ma H., Xie Q. (2016). Response-guided peginterferon therapy in patients with HBeAg-positive chronic hepatitis B: A randomized controlled study. *Journal of Hepatology*.

[10] Xie Q., Zhou H., Bai X. (2014). A randomized, open-label clinical study of combined pegylated interferon alfa-2a (40KD) and entecavir treatment for hepatitis B ‘e’ antigen-positive chronic hepatitis B. *Clinical Infectious Diseases*.

[11] Liu Y.-H., Wu T., Sun N. (2014). Combination therapy with pegylated interferon alpha-2b and adefovir dipivoxil in HBeAg-positive chronic hepatitis B versus interferon alone: A prospective, randomized study. *Journal of Huazhong University of Science and Technology (Medical Sciences)*.

[12] Lau G. K. K., Piratvisuth T., Kang X. L. (2005). Peginterferon Alfa-2a, lamivudine, and the combination for HBeAg-positive chronic hepatitis B. *The New England Journal of Medicine*.

[13] Chan H. L., Leung N. W., Hui A. Y. (2005). A Randomized, Controlled Trial of Combination Therapy for Chronic Hepatitis B: Comparing Pegylated Interferon-*α*2b and Lamivudine with Lamivudine Alone. *Annals of Internal Medicine*.

[14] Janssen H. L. A., Van Zonneveld M., Senturk H. (2005). Pegylated interferon alfa-2b alone or in combination with lamivudine for HBeAg-positive chronic hepatitis B: a randomised trial. *The Lancet*.

[15] Jadad A. R., Moore R. A., Carroll D. (1996). Assessing the quality of reports of randomized clinical trials: Is blinding necessary?. *Controlled Clinical Trials*.

[16] Sweeting M. J., Sutton A. J., Lambert P. C. (2004). What to add to nothing? Use and avoidance of continuity corrections in meta-analysis of sparse data. *Statistics in Medicine*.

[17] Wetterslev J., Thorlund K., Brok J., Gluud C. (2009). Estimating required information size by quantifying diversity in random-effects model meta-analyses. *BMC Medical Research Methodology*.

[18] Govan L., Wu O., Xin Y., Hutchinson S. J., Hawkins N. (2015). Comparative effectiveness of antiviral treatment for hepatitis B: a systematic review and Bayesian network meta-analysis. *European Journal of Gastroenterology & Hepatology*.

[19] Njei B., Gupta N., Ewelukwa O., Ditah I., Foma M., Lim J. K. (2016). Comparative efficacy of antiviral therapy in preventing vertical transmission of hepatitis B: A network meta-analysis. *Liver International*.

[20] Marcellin P., Ahn S. H., Chuang W.-L. (2016). Predictors of response to tenofovir disoproxil fumarate plus peginterferon alfa-2a combination therapy for chronic hepatitis B. *Alimentary Pharmacology & Therapeutics*.

[21] Coffin C. S., Lee S. S. (2015). New paradigms in hepatitis B management: Only diamonds are forever. *British Medical Bulletin*.

[22] Yuen M.-F., Wong D. K.-H., Sum S. S.-M. (2005). Effect of lamivudine therapy on the serum covalently closed-circular (ccc) DNA of chronic hepatitis B infection. *American Journal of Gastroenterology*.

[23] Sonneveld M. J., Rijckborst V., Boucher C. A. B., Hansen B. E., Janssen H. L. A. (2010). Prediction of sustained response to peginterferon alfa-2b for hepatitis B e antigen-positive chronic hepatitis B using on-treatment hepatitis B surface antigen decline. *Hepatology*.

[24] Osiowy C., Coffin C., Andonov A. (2016). Review of laboratory tests used in monitoring hepatitis b response to pegylated interferon and nucleos(t)ide analog therapy. *Current Treatment Options in Infectious Diseases*.

